# Editorial: Multidisciplinary approaches to enhance healing in biofilm-infected chronic wounds

**DOI:** 10.3389/fcimb.2026.1986348

**Published:** 2026-09-23

**Authors:** Danica Z. Zmejkoski, Dijana Mitic, Jelena Carkic, Nemanja Zdravkovic

**Affiliations:** 1 VINČA Institute of Nuclear Sciences - National Institute of the Republic of Serbia, Belgrade, Serbia; 2 Faculty of Dental Medicine, University of Belgrade, Belgrade, Serbia; 3 Scientific Institute for Veterinary Medicine, Belgrade, Serbia

**Keywords:** antimicrobial nanoparticle dressings, biofilm disruption, ECM-mimicking hydrogels, stimuli-responsive biomaterials, synergistic regenerative therapies

Chronic wounds remain one of the most pressing and complex challenges in modern healthcare. Conditions such as diabetic foot ulcers, pressure injuries, and venous leg ulcers impose a substantial burden on patients and healthcare systems, often leading to prolonged suffering, recurrent infections, and, in severe cases, limb amputation. A defining feature of these wounds is their inability to progress through the normal stages of healing, frequently becoming trapped in a persistent inflammatory state driven by microbial colonization and biofilm formation.

Biofilms, structured communities of microorganisms embedded in a self-produced extracellular matrix, play a central role in chronic wound pathology. These polymicrobial aggregates exhibit heightened tolerance to antimicrobial agents and host immune responses, thereby sustaining infection and delaying tissue repair. The complexity of host–microbe interactions within the wound microenvironment further complicates treatment, as biofilms not only protect pathogenic bacteria but also modulate inflammation, angiogenesis, and cellular proliferation. Consequently, effective management of chronic wounds requires strategies that simultaneously disrupt biofilms and promote regenerative processes.

This Research Topic brings together a diverse collection of studies that reflect the inherently multidisciplinary nature of this field, spanning microbiology, biomaterials science, clinical medicine, and bioengineering. The contributions highlight a paradigm shift from conventional wound care approaches to integrated therapeutic platforms that address both infection control and tissue regeneration.

Several articles focus on developing advanced biomaterials as multifunctional therapeutic platforms. Yang et al. demonstrate that catechol-modified chitosan/PEG hydrogels combining intrinsic antibacterial activity with sustained TGF-β3 delivery promote angiogenesis, enhance cellular proliferation, and accelerate wound closure. Likewise, Rojczyk et al. report improved healing outcomes using hydrogel-based dressings containing octenidine, emphasizing the potential of multifunctional biomaterials to actively modulate the wound microenvironment while controlling infection.

Advances in molecular diagnostics are also reshaping our understanding of chronic wound infections. Using metagenomic and transcriptomic approaches, Diaz-Velis et al. reveal the remarkable diversity of chronic wound microbiota, including previously underrecognized microorganisms that may contribute to biofilm persistence. These findings emphasize the value of integrating molecular profiling into clinical practice to improve pathogen detection and support more personalized treatment strategies.

Beyond pathogen identification, modulation of the wound microbiome itself is emerging as a promising therapeutic approach. Qiu et al. used 16S rRNA sequencing to investigate the effects of Golden Wanhong Ointment in patients with diabetic foot ulcers and found that treatment reshaped the wound microbial community, reduced the abundance of infection-associated genera such as Prevotella and Escherichia–Shigella, lowered inflammatory markers including IL-6 and TNF-α, and improved clinical healing outcomes. These findings further support the concept that targeted microbiome modulation can create a more favorable environment for tissue repair.

The interplay between microorganisms and host immune responses is another recurring theme in this Research Topic. Understanding these interactions is essential for developing therapies that not only eliminate infection but also restore the balance required for successful healing. In this context, Hu et al. review the emerging application of electrets, materials capable of generating stable electric fields, as a novel therapeutic platform. Owing to their biocompatibility, low cost, and long-lasting activity, electrets have shown potential to accelerate tissue regeneration, reduce infection, and even enable real-time wound monitoring, although further translational research is still needed.

Several contributions also evaluate advanced clinical interventions for chronic wound management. Guan et al. summarize evidence from systematic reviews and meta-analyses comparing therapies such as negative pressure wound therapy (NPWT/VAC), mesenchymal stem cell-based treatments, artificial skin substitutes, extracorporeal shockwave therapy (ESWT), and platelet-rich plasma (PRP). While these approaches generally outperform standard care, the authors highlight the need for larger, well-designed clinical trials to establish their long-term efficacy. Similarly, Zheng et al. discuss the benefits of combining NPWT with gas therapy and advanced wound dressings, whereas Cui et al. demonstrate that targeted antiseptic therapies may accelerate healing and improve recovery in critically ill patients without increasing adverse procedures.

Other studies broaden our understanding of factors that influence chronic wound pathology. Zhu et al. show that foreign bodies can serve as substrates for bacterial adhesion and biofilm formation, with material properties directly affecting microbial behavior. Their findings emphasize the importance of appropriate material selection, timely surgical intervention, and effective wound debridement. Complementing these observations, Reynoso et al. explore endocrine and molecular signaling pathways involved in wound healing, highlighting opportunities for biomarker-guided and personalized therapeutic strategies.

Finally, Zhang et al. review microbiotherapy as an emerging adjunctive approach for burn wound management. Their work discusses the therapeutic potential of probiotics, bacteriophages, and microbial metabolites to suppress pathogenic microorganisms, restore microbial and immune homeostasis, and promote tissue repair, further illustrating the growing interest in microbiome-based therapies for chronic and complex wounds.

Collectively, the studies presented in this Research Topic demonstrate that successful management of biofilm-infected chronic wounds requires an integrated approach that combines innovative biomaterials, advanced molecular diagnostics, microbiome-targeted interventions, and evidence-based clinical therapies (**Figure 1**). Together, these multidisciplinary efforts are expanding our understanding of chronic wound biology and providing a foundation for developing more effective, personalized, and sustainable treatment strategies.

**Figure 1:**
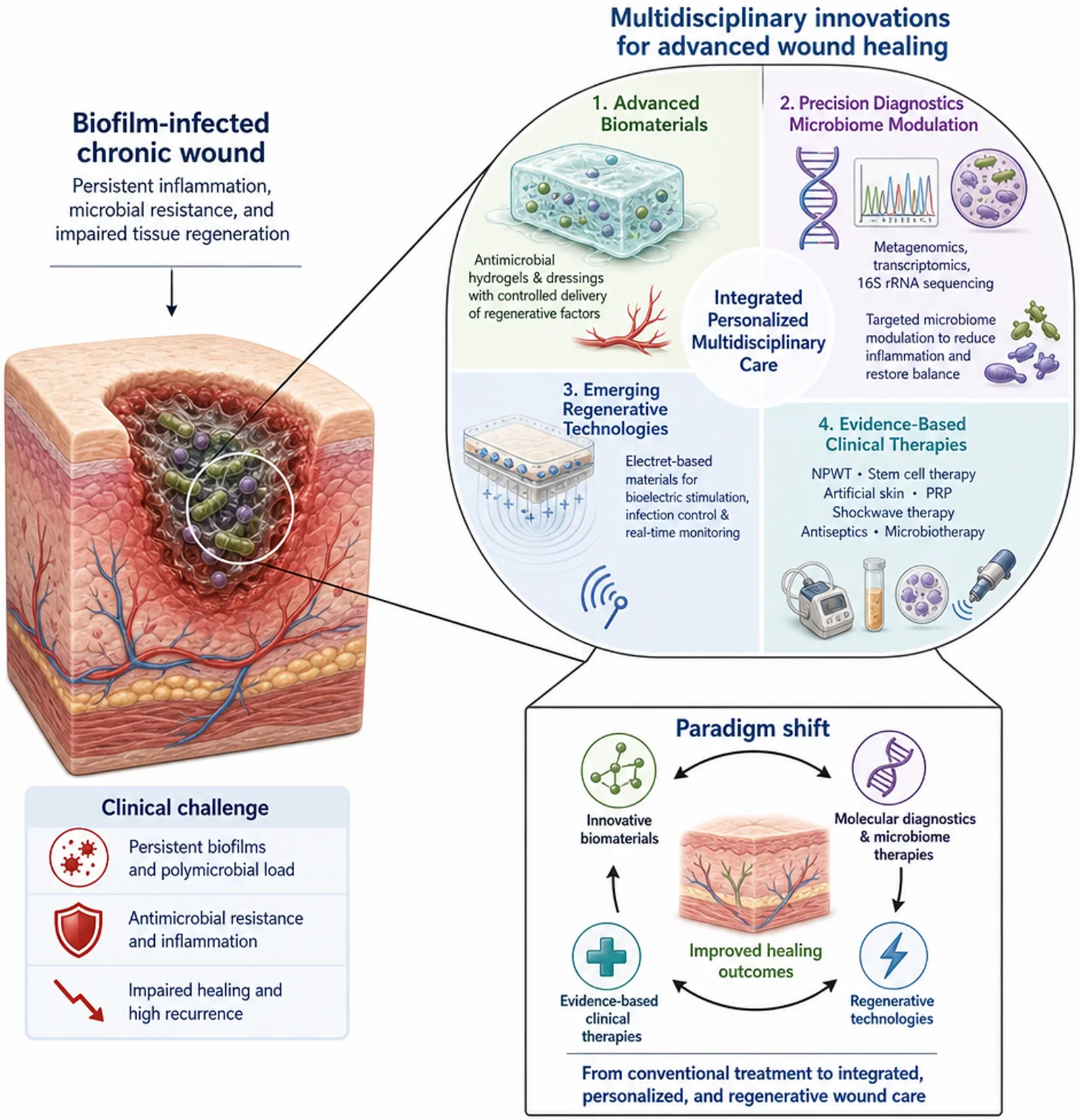
From complex wound biology to personalized care. Schematic representation of the complex chronic wound microenvironment, characterized by persistent inflammation, microbial biofilm formation, and impaired tissue repair. The figure highlights four complementary innovation pillars: advanced biomaterials, precision diagnostics, regenerative technologies, and evidence-based therapies toward a more personalized and effective approach to chronic wound care.

